# Acceptability, Feasibility, Drug Safety, and Effectiveness of a Pilot Mass Drug Administration with a Single Round of Sulfadoxine–Pyrimethamine Plus Primaquine and Indoor Residual Spraying in Communities with Malaria Transmission in Haiti, 2018

**DOI:** 10.4269/ajtmh.22-0623

**Published:** 2023-05-09

**Authors:** Michelle A. Chang, Daniel Impoinvil, Karen E. S. Hamre, Paul-Emile Dalexis, Jean-Baptiste Mérilien, Amber M. Dismer, Bernadette Fouché, Luccene Desir, Kathleen Holmes, Willy Lafortune, Camelia Herman, Eric Rogier, Gregory S. Noland, Alyssa J. Young, Thomas Druetz, Ruth Ashton, Thomas P. Eisele, Justin Cohen, Lotus van den Hoogen, Gillian Stresman, Chris Drakeley, Emilie Pothin, Ewan Cameron, Katherine E. Battle, John Williamson, Marc-Aurèle Telfort, Jean Frantz Lemoine

**Affiliations:** ^1^ Malaria Branch, Center for Global Health, U.S. Centers for Disease Control and Prevention, Atlanta, Georgia;; ^2^ Entomology Branch, Center for Global Health, U.S. Centers for Disease Control and Prevention, Atlanta, Georgia;; ^3^ CDC Foundation, Atlanta, Georgia;; ^4^ IMA World Health, Port-au-Prince, Haiti;; ^5^ Programme National de Contrôle de la Malaria, Ministère de la Santé Publique et de la Population, Port-au-Prince, Haiti;; ^6^ Emergency Response and Recovery Branch, Center for Global Health, U.S. Centers for Disease Control and Prevention, Atlanta, Georgia;; ^7^ The Carter Center, Atlanta, Georgia;; ^8^ Center for Applied Malaria Research and Evaluation, Tulane University School of Public Health and Tropical Medicine, New Orleans, Louisiana;; ^9^ Clinton Health Access Initiative, Washington, District of Columbia;; ^10^ London School of Hygiene & Tropical Medicine, London, United Kingdom;; ^11^ Swiss Tropical and Public Health Institute, Basel, Switzerland;; ^12^ University of Basel, Basel, Switzerland;; ^13^ School of Public Health, Curtin University, Bentley, Australia;; ^14^ Institute for Disease Modeling, Bill & Melinda Gates Foundation, Seattle, Washington

## Abstract

For a malaria elimination strategy, Haiti’s National Malaria Control Program piloted a mass drug administration (MDA) with indoor residual spraying (IRS) in 12 high-transmission areas across five communes after implementing community case management and strengthened surveillance. The MDA distributed sulfadoxine–pyrimethamine and single low-dose primaquine to eligible residents during house visits. The IRS campaign applied pirimiphos–methyl insecticide on walls of eligible houses. Pre- and post-campaign cross-sectional surveys were conducted to assess acceptability, feasibility, drug safety, and effectiveness of the combined interventions. Stated acceptability for MDA before the campaign was 99.2%; MDA coverage estimated at 10 weeks post-campaign was 89.6%. Similarly, stated acceptability of IRS at baseline was 99.9%; however, household IRS coverage was 48.9% because of the high number of ineligible houses. Effectiveness measured by *Plasmodium falciparum* prevalence at baseline and 10 weeks post-campaign were similar: 1.31% versus 1.43%, respectively. Prevalence of serological markers were similar at 10 weeks post-campaign compared with baseline, and increased at 6 months. No severe adverse events associated with the MDA were identified in the pilot; there were severe adverse events in a separate, subsequent campaign. Both MDA and IRS are acceptable and feasible interventions in Haiti. Although a significant impact of a single round of MDA/IRS on malaria transmission was not found using a standard pre- and post-intervention comparison, it is possible there was blunting of the peak transmission. Seasonal malaria transmission patterns, suboptimal IRS coverage, and low baseline parasitemia may have limited the effectiveness or the ability to measure effectiveness.

## INTRODUCTION

The Republic of Haiti, along with the bordering country of the Dominican Republic, are the only countries in the Caribbean with endemic malaria transmission. The two countries (population, 22,250,433) comprise the island of Hispaniola and report malaria caused almost exclusively by *Plasmodium falciparum,* with 39,097 cases of *P. falciparum *malaria reported in 2020.^1^^,^^2^ Similar to previous years, 97% of the reported malaria cases in Hispaniola were from Haiti, and more than 57% of those were localized in communities in two contiguous departments (Grand’Anse and Sud) of 10 in Haiti.^3^ Most of the smaller, eastern Caribbean islands eliminated malaria during the Global Malaria Eradication Program of the 1950s and 1960s by deploying indoor residual spraying (IRS) with dichlorodiphenyltrichloroethane (DDT), mass drug administration (MDA) campaigns using chloroquine (CQ), and environmental modifications.^4^^,^^5^ In 1966, Haiti successfully reached low malaria levels with a slide positivity of < 0.1% in high-transmission areas using similar strategies—namely, large-scale MDA with CQ plus pyrimethamine, and IRS with DDT.^4^ By 1972, the Global Malaria Eradication Program ended with the development of parasite and vector resistance to pyrimethamine and DDT, respectively, along with waning donor funding.^4^^,^^6^ Since then, Haiti’s National Malaria Control Program (NMCP) has experienced periods of peak numbers of cases, such as seen in 2011 with approximately 34,350 cases and a slide positivity of 18.6%, reflecting loss of control at that time.^1^^,^^7^

In the past decade, MDA received renewed interest from the global malaria community and donors. Multiple randomized, controlled trials have found that MDA rapidly reduces parasite prevalence, and thus could be useful in accelerating malaria elimination in low-transmission countries.^8^^–^^11^ Based on the early results of the trials, the Malaria Policy Advisory Committee to the WHO recommended in 2015 that MDA could be considered in pre-elimination settings where surveillance, case management, and vector control were implemented and accessible.^12^

No recent MDA campaign has used solely a single-dose treatment for malaria elimination because most antimalarial medications require a multiple-day regimen. Although sulfadoxine–pyrimethamine (SP) is administered as a one-dose treatment, there is widespread resistance of the *P. falciparum *parasite to this drug. Haiti is one of the few countries where the *P. falciparum *parasite remains susceptible to SP.^13^ A one-time, single-encounter medication deployed in an MDA campaign could improve acceptability, adherence, and, ultimately, coverage and effectiveness of the intervention. As part of a package of interventions for malaria elimination, implemented by the NMCP with support from Malaria Zero, a house-to-house pilot campaign using one round of MDA with SP plus a single low dose (SLD) of primaquine (PQ) and IRS with pirimiphos–methyl was conducted in selected communities in 2018. The objective was to assess the acceptability and feasibility of MDA and IRS, because neither intervention had been deployed in Haiti in recent decades. In addition, the NMCP sought to assess the campaign’s effectiveness toward malaria elimination under optimal programmatic conditions and using novel implementation and assessment tools. In the programmatic context, effectiveness meant interrupting malaria transmission temporarily (1–3 months) or decreasing (by > 15% change) parasite prevalence in the treated communities. The development of the campaign tools, deployment/monitoring systems, and the results of the study were intended to inform the country’s malaria elimination strategy. Multiple future campaigns would be needed to reach malaria elimination.

In line with the WHO’s recommendation, the MDA/IRS campaign was implemented in 2018 along with strengthening malaria surveillance, case management, and entomological monitoring capacity. This article focuses on the results of the baseline and three follow-up surveys for the MDA/IRS component of the package of interventions. The study was part of the Malaria Zero project to support Haiti and the Dominican Republic in accelerating elimination of malaria from the island of Hispaniola.

## METHODS

### Setting.

**Figure 1.**
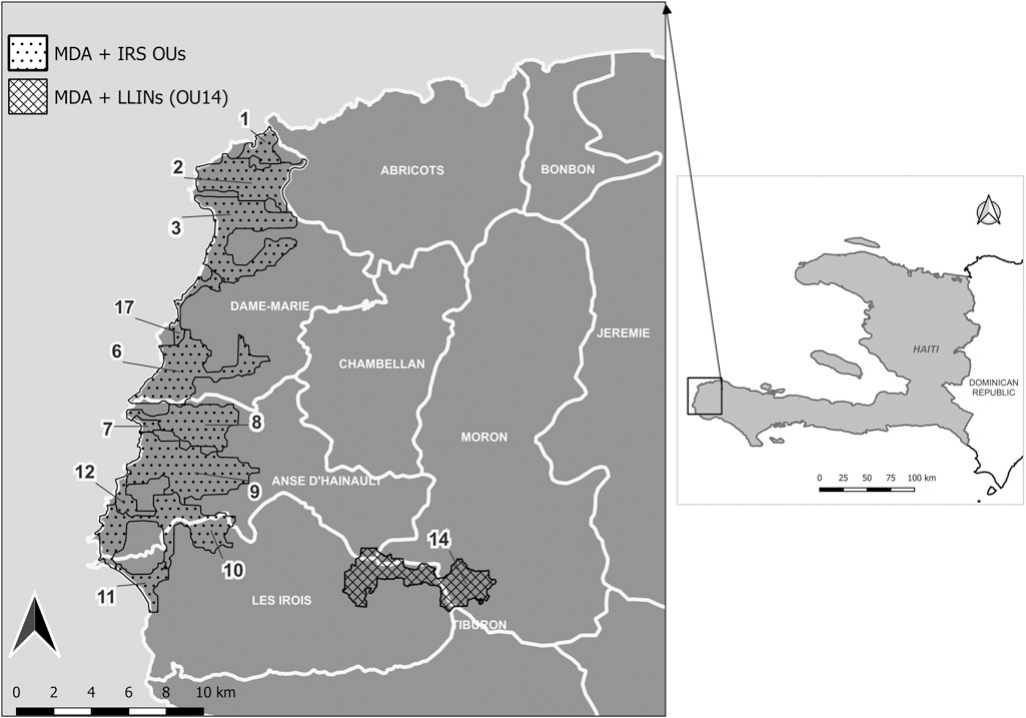
Map of operational units (OUs) that received mass drug administration (MDA), indoor residual spraying (IRS), or long-lasting insecticide-treated nets (LLINs) during the 2018 pilot campaign in Grand’Anse, Haiti. The OU numbers are not consecutive in this figure.

This pilot was conducted in the Grand’Anse Department located on the southern peninsula of Haiti. The MDA/IRS campaign was implemented in selected communities of five communes (Anse d’Hainault, Chambellan, Dame Marie, Les Irois, and Moron; population estimate, 156,000) in the Grand’Anse Department, where typically > 50% of Haiti’s malaria cases have been reported.^14^ The terrain is coastal, with inland forests and steep mountains reaching peaks of more than 2,300 m separated by narrow valleys. Except for one operational unit (OU; defined in “Study design”), all the intervention communities reside at coastal or lower elevations (Figure 1). Rainfall occurs mainly during two seasons: the primary rains from October through December that typically peak in November, and a secondary rainy season with a peak rainfall in May.^15^ Accordingly, the peak malaria transmission season follows the rainfall and is usually seen from October through January.^16^

Routine malaria control program interventions have been implemented nationally under Haiti’s Global Fund (GF) malaria grants since 2003. The GF grants have supported aggregated malaria case reporting through the national surveillance system, free access to malaria testing with histidine-rich protein 2-based (HRP2) rapid diagnostic tests (RDTs), treatment with CQ plus a single dose of primaquine PQ, larval source management, and the distribution of long-lasting insecticide-treated nets (LLINs).^17^^,^^18^ With the support of Malaria Zero starting in 2015, the malaria program activities in Grand’Anse were strengthened by 1) transitioning malaria surveillance to individual case-based reporting (began November 2017), 2) implementing community case management of malaria in communities at least 5 km away from health services (began September 2018), 3) increasing community awareness/knowledge of malaria and community participation in malaria control and elimination interventions (September 2018), and 4) training to improve larvicide (*Bacillus thuringiensis israelensis*) application (December 2018). In addition, an LLIN campaign was conducted in Grand’Anse in October 2017 under Haiti’s GF grant to distribute two LLINs per household through community fixed posts.

### Study design.

Communities with high malaria transmission (relative to other communities in Haiti) were identified based on surveillance data, modeled predictions of malaria transmission [malaria basic reproductive rate under control (Rc)], and a rapid assessment of parasite prevalence and seroprevalence, which have been described elsewhere^16^ (K. E. S. H., manuscript in preparation). The communities with a predicted Rc in the top quartile with evidence of ongoing transmission (based on malaria case surveillance; seroprevalence of recent exposure) were selected.^19^ The study area was segmented into 12 OUs that served as the enumeration areas for sampling (Figure 1). The OUs were geographic areas with borders drawn to group similar predicted Rc values within each unit. Each OU was assumed to have homogeneous transmission within its limited geographic size (average size, 3.3 km^2^). In July 2018, all residents in the study area were censused and houses were georeferenced. The census provided the sampling frame for households. The study used an observational, cross-sectional design to assess parasite prevalence, malaria seroprevalence, and sociobehavioral factors before the MDA/IRS campaign (baseline) and then post-campaign planned at 6 weeks, 6 months, and 12 months. (The first and third post-campaign surveys were delayed and are described in “Results.”) The time points for the surveys were selected for the following reasons: 1) at baseline (T0) data collection scheduled at the beginning of malaria transmission season before the MDA/IRS campaign, 2) at 6 weeks (T1) to evaluate the effect of the campaign that is expected within 1 to 3 months, 3) at 6 months (T2) to evaluate any extended effect during the low-transmission season and to assess seroprevalence changes using short- and mid-term antibody markers, and 4) at 12 months (T3) to obtain a comparison at the same transmission season as baseline and assess seroprevalence changes using longer term antibody markers.^11^

The sample size per survey was calculated using the following parameters: 80% statistical power, 5% chance of a type I error (two sided), and an intracluster correlation coefficient of 0.1277 based on prior study data. A sample size of 1,250 individuals for each survey would detect a 15% decrease in parasite prevalence from baseline to the follow-up time point. Based on previous surveys, the average household size was expected to be approximately 3.5 persons. With an estimated survey refusal rate of at least 4%, 35 households were targeted for sampling per OU.^20^ All household members were asked to participate. Because of the limited number of clusters and high intracluster correlation coefficient, it would not be possible to detect differences in parasite prevalence between baseline and follow-up with a baseline prevalence < 15% or a smaller detectable difference. Households were selected by simple random sampling from the roster of those identified by the census.

### Pilot MDA/IRS campaign.

**Figure 2.**
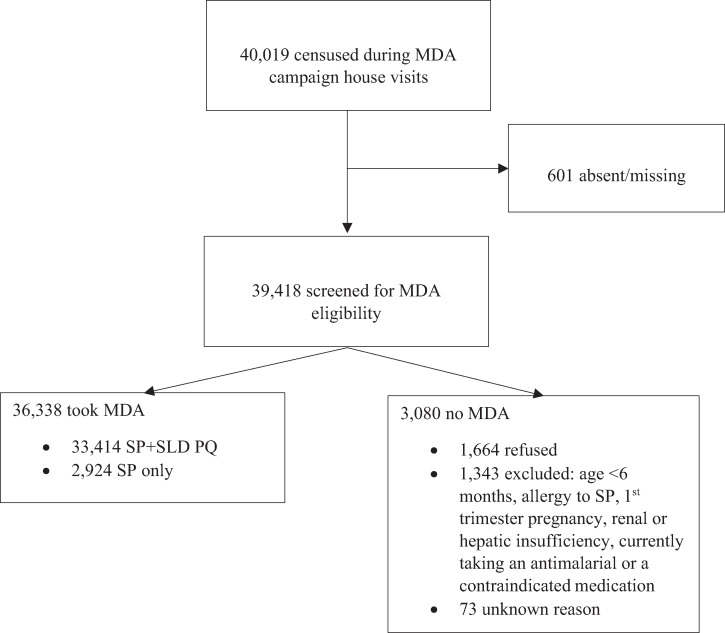
Flowchart of individuals censused and screened for mass drug administration (MDA) eligibility, and who took sulfadoxine–pyrimethamine (SP) and/or primaquine (PQ). The reasons for not participating in MDA include absenteeism/unavailability despite return visits, refusals, and being ineligible based on the inclusion criteria or medical screening. These data were collected during the campaign as part of the monitoring system. SLD = single low dose.

The intervention was a combined house-to-house MDA and IRS campaign in communities with a total population of 40,019 people. Both interventions were preceded by community engagement activities. From September 3 to 14, 2018, training sessions were conducted on malaria interventions and their health benefits with Community Health Councils (CHCs), which were voluntary community leadership groups organized as part of the Malaria Zero project. Prior to and during the MDA and IRS campaigns, CHCs mobilized their communities during weekly outreach activities using microphones, church and school meetings, door-to-door outreach, and public announcements. Radio broadcasts ran from August 28 to November 15, 2018 on nine local stations, banners were set up in high-visibility locations, and sound trucks were deployed just prior to each campaign. Campaign staff worked with the CHCs throughout the campaign to conduct outreach and to address community questions and concerns. The MDA component of the campaign was implemented from October 10 to November 6, 2018 and was coordinated with IRS implementation. The selection of SP for the MDA was based on the *P. falciparum *parasite susceptibility profile in Haiti, the global experience with the use of SP in seasonal malaria chemoprophylaxis campaigns, intermittent preventive treatment in pregnant women, preferred operational aspects for mass administration (single-dose regimen), and consensus across partners, with the final decision made by the Haitian Ministry of Health. An SLD of 0.25 mg/kg PQ (maximum, 15 mg) was included in the MDA regimen for the gametocytocidal effect to decrease parasite transmission from existing infections at the time of the campaign.^21^^–^^23^ The eligibility criteria for participation in the MDA campaign included the provision of voluntary consent, age of 6 months or older, and no known allergy to sulfonamides. A medical screening questionnaire was administered to each participant to exclude those who were currently taking a sulfonamide, an antimalarial medication, or any medication contraindicated to be co-administered with SP; women in their first trimester of pregnancy or unknown pregnancy status; or anyone who reported renal or hepatic insufficiency. Additional questionnaire screening to assess eligibility for PQ was conducted. Those who were currently taking PQ or a medication contraindicated for co-administration, pregnant, breastfeeding, or had a known allergy to PQ were administered SP only. Women of reproductive age (15–49 years) were assessed for pregnancy by questionnaire and urine human chorionic gonadotropin testing. The dosing of medicines was based on weight-associated age categories that have been in use in Haiti for SP (25/1.25–33/1.7 mg/kg; maximum,1,500/75 mg). Medications were administered under direct observation during the household visit. Members of the household who were absent during the initial visit were scheduled to meet at a designated follow-up post or the team returned to the house. Any member of the household who was traveling at the time of the campaign, and who would return within 30 days of the initial visit, would be scheduled for a visit by the MDA team. All screening and follow-up action algorithms, dosing tables, and data collection instruments were programmed into the CommCare (Dimagi, Inc., Cambridge, MA) platform for mobile data collection. Based on the campaign monitoring data (not the surveys), the total number of people treated with SP with or without SLD PQ was 36,338—a 90.8% effective coverage. The denominator for calculating the effective coverage included all individuals who were censused during the household visits, including those who were ultimately ineligible/excluded, absent, or refused (Figure 2).

Pharmacovigilance (PV) using the WHO definitions for monitoring and reporting adverse events (AEs) was initiated during the first week of the MDA component of the campaign.^24^ The PV system continued 30 days beyond the last day of the MDA campaign. A nurse at each of the eight health facilities that served the communities receiving MDA was trained to complete a standardized AE reporting form, treat patients based on their symptoms, and alert the field manager to the AE. Moderate or severe AEs were evaluated by an NMCP staff physician or program coordinator. The PV reports were reviewed by two study physicians to resolve discrepancies and to confirm the final classification of AE severity and relationship with SP and PQ.

Although the main malaria vector, *Anopheles albimanus*, tends to be exophagic and exophilic, these characteristics are not exclusive.^15^ Prior studies and experience have shown that IRS in Haiti can be effective.^4^ The IRS component was conducted from October 15 to November 2, 2018 by the NMCP, the Grand’Anse municipal public health office, and Abt Associates. A spray target of approximately 9,497 potentially eligible households was set, taking into consideration site accessibility and structure eligibility. Houses constructed of the following were eligible for IRS: bamboo, thatch, mud, brick, cement or concrete, wood (lumber), and sticks. Houses constructed of sackcloth, textured fabric, corrugated iron, or plastic sheeting were not eligible for IRS. The IRS campaign was implemented using the organophosphate insecticide pirimiphos–methyl (Actellic 300CS), to which no resistance has been detected in the *A. albimanus* population.^25^ In 11 of the 12 OUs along the coast, IRS was conducted; the inland OU 14 was not included because of the challenging terrain (Figure 1). Rather, all households in OU 14 received one LLIN for every two people during a house-to-house distribution from January 19 to 23, 2019. The IRS campaign included advance geographic reconnaissance of the intervention area, recruitment and training of local spray operators to promote community acceptance, environmental compliance assessments, and micro-planning meetings with local Ministry of Health officials and community leaders. Global standards for best management practices for IRS campaigns were followed.^26^^,^^27^

### Survey participants.

All residents of the sampled households were provided information on the risks and benefits of participating in the survey before seeking consent. Adults (≥ 18 years old) who consented were eligible to participate. Children and minors (< 18 years old) were eligible if parents or guardians consented. In addition, assent was sought from minors (7–17 years old). If family members were absent during the first visit, up to two additional visits to the household were made within a 3-day window to meet the absent members.

### Survey variables and biomarkers.

Standardized survey questionnaires were programmed into the CommCare platform and loaded onto tablets. The residence location, GPS coordinates, altitude, and household member composition were collected. Household-level questions were posed to the head of household or primary caregiver to assess attitudes and acceptability toward MDA, IRS, and the goal of malaria elimination. Additional variables collected at the household level included operational aspects of the intervention delivery by campaign teams, awareness of the campaign, and household-level ownership of LLINs. Individual-level variables captured demographics, whether the medications were ingested, attitude toward MDA, AEs, and reasons for not taking the MDA medication.

A finger-prick blood sample was collected from each consenting participant to test for the presence of *P. falciparum *parasites by a conventional RDT (SD Bioline Malaria Antigen P.f., Standard Diagnostics Inc., Republic of Korea) and a highly sensitive RDT (hsRDT) (Alere Malaria Ag P.f., Standard Diagnostics), and to prepare dried blood spots on filter papers (Whatman 903 Protein Saver Card, GE Healthcare, Chicago, IL). If either the RDT or hsRDT was positive, the malaria parasitemia status was considered positive for the analysis. Studies show a modest advantage of hsRDTs for detecting low-density parasitemia compared with RDTs, especially in asymptomatic populations and could aid in identifying more infections.^28^ The dried blood spots were air-dried, then stored in an air-tight bag with desiccant to be analyzed for the presence of HRP2 and serological markers of exposure to *Plasmodium*. Detection of HRP2 antigen by multiplex laboratory assay was performed as described previously.^29^ The serological assays for the early transcribed membrane protein 5 antigen 1 (Etramp 5 ag 1), apical membrane antigen 1 (AMA1), and merozoite surface protein 1 (MSP1) were conducted at the CDC in Atlanta, GA.^30^^,^^31^ Seropositivity to either AMA1 or MSP1 (AMA1/MSP1) was used as a combined variable for analysis to increase sensitivity of identifying previous long-term (a few years) *P. falciparum *exposure, whereas Etramp 5 ag 1 was selected to represent recent (6 months) infection with *P. falciparum*.^30^^,^^32^^,^^33^

### Data sources and statistical methods.

Multiple data sources were used in this analysis. The primary data source was the pre- and post-campaign household surveys with the collection of biomarkers. Additional data sources included the campaign monitoring data, PV reports of AEs associated with the MDA campaign, the pre-survey census, MDA and IRS campaign monitoring data, and insecticide wall bioassays. Analyses, accounting for clustering at the OU level, were conducted in SAS version 9.4 (SAS Institute, Cary, NC). For calculating point estimates and their corresponding 95% confidence limit (CL), sampling weights were based on the probability of selecting the household in each of the 12 OUs. Empirically estimated SEs were used to account for the correlation of individual data within households.

## RESULTS

### Survey and participant characteristics.

**Figure 3.**
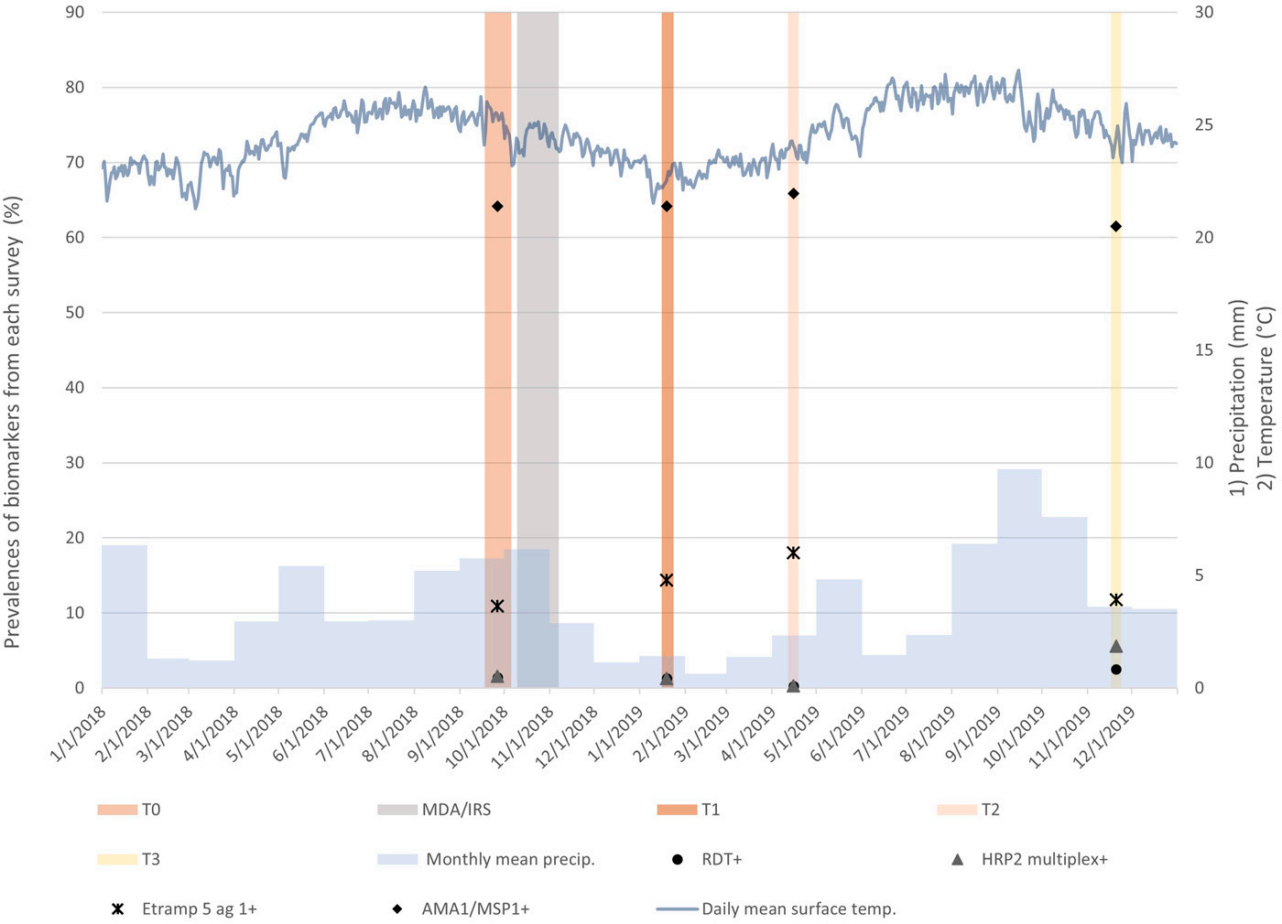
Timing of surveys and the mass drug administration (MDA)/indoor residual spraying (IRS) campaign, parasite and antibody response prevalence at different time points (T0, baseline; T1, 10 weeks; T2, 6 months; and T3, 14 months) relative to the mean daily precipitation (precip.) and surface temperature (temp.) in Grand’Anse. The indicator bands for the surveys and the MDA/IRS campaign correspond to the actual dates of the fieldwork. Because of large daily variations, precipitation is displayed as the monthly mean to visualize the trend better. Climate data were obtained from the National Aeronautics and Space Administration’s Giovanni interface (https://giovanni.gsfc.nasa.gov/giovanni), a Web-based platform for the visualization and analysis of climate data. For the rainfall data, the Global Precipitation Model at 0.1° spatial resolution was used; for the temperature data, the Global Land Data Assimilation System Version 2 at 0.25° spatial resolution was used. + = presence of antigen or antibody indicating positive test result; AMA1 = apical membrane antigen 1; Etramp 5 ag1 = early transcribed membrane protein 5 antigen 1; HRP2 = histidine-rich protein 2; MSP1 = merozoite surface protein 1; RDT = rapid diagnostic test.

Main characteristics of the baseline and three follow-up surveys are presented in Table 1. The first follow-up survey (T1) and the final follow-up survey (T3) were delayed from 6 weeks to 10 weeks and 12 months to 14 months, respectively, by logistical challenges and intermittent political insecurity. The rate of participation for the surveys and blood tests was less than expected, resulting in a slightly smaller sample obtained for the T0, T2, and T3 surveys. Sampled houses and participant characteristics were similar across surveys; however, the proportions of individuals who completed the survey and RDT were less in T2 and T3 compared with T0. The mean daily precipitation and temperature varied across the survey time points and correspond with the seasonal pattern of malaria transmission in Haiti (Figure 3).

**Table 1:** Survey and participant characteristics

Characteristic	Pre-campaign	Post-campaign		
	Baseline survey (T0)	Survey 1 (T1)	Survey 2 (T2)	Survey 3 (T3)
Dates of data collection	September 18–October 5, 2018	January 16–23, 2019	April 12–18, 2019	November 17–23, 2019
Seasonality	Begin high transmission	End high transmission	Low transmission	High transmission
Mean daily precipitation, mm (range)*	10.5 (0.25–47.5)	1.0 (0.4–1.7)	0.9 (0.3–1.3)	7.2 (0.004–47.6)
Mean daily surface temperature, °C (range)*	25.2 (24.1–26.0)	22.7 (22.2–23.2)	24.0 (23.5–24.3)	24.2 (23.5–25.0)
Sample size, *n*	1,344	1,511	1,524	1,306
No. of households	392	417	381	368
Female, *n* (%)†	717 (53.4)	838 (55.5), *P* = 0.26	790 (51.8), *P* = 0.42	706 (54.1), *P* = 0.71
Median age, years (IQR)†	24 (10–45)	23 (9–43), *P* = 0.34	22 (10–45), *P* = 0.98	23 (9–43), *P* = 0.54
Median household size, *n *(IQR)	4 (3–6)	4 (3–5)	5 (3–7)	4 (3–6)
Completed survey and RDT, *n *(%)†‡	1,196 (89.0)	1,363 (90.2), *P* = 0.24	1,221 (80.1), *P* < 0.0001	1,122 (85.9), *P* = 0.02

IQR = interquartile range; RDT = rapid diagnostic test.

* Climate data were obtained from the National Aeronautics and Space Administration’s Giovanni interface (https://giovanni.gsfc.nasa.gov/giovanni), a Web-based platform for the visualization and analysis of climate data. For the rainfall data, the Global Precipitation Model at 0.1° spatial resolution was used; for the temperature data, the Global Land Data Assimilation System Version 2 at 0.25° spatial resolution was used.

† *P* value for χ^2^ test compared with T0.

‡ Percentage of total sample.

### Acceptability: coverage indicators of the MDA/IRS campaign.

Prior to the campaign, the stated acceptability of MDA and IRS was high at T0, when the primary respondents of the households were asked whether they would take medications or allow spraying in their home for malaria elimination—nearly all responded yes (98.9% and 99.9%, respectively). This reflected the expected willingness of the head of household or primary caregiver to accept the interventions delivered by the campaign, and thereby reach individual household members (Table 2).

**Table 2:** Indicators of acceptability and coverage of MDA and IRS

Indicator	Time point, % (95% CL)			
	Baseline (T0)	Survey 1 (T1)	Survey 2 (T2)	Survey 3 (T3)
HH willing to take medications for malaria elimination campaign, head of HH response	99.2 (98.2–100)	–	–	–
Individuals who took MDA medications	–	89.6 (87.3–92.0)	86.9 (83.4–90.4)	74.9 (69.9–79.2)
Individuals who would take MDA again among those who took medications during campaign	–	99.6 (99.2–100)	–	–
HH willing to accept IRS for malaria elimination campaign	99.9 (99.8–100)	–	–	–
HH was sprayed (no OU 14)	–	48.9 (41.5–56.3)	60.6 (53.0–68.1)	52.6 (45.3–59.9)
HH that would accept IRS again among those that had IRS during campaign	–	85.0 (80.3–89.7)	–	–
HH owns any bed net	3.2 (0–9.8), OU 14 only	11.4 (0.3–22.5), OU 14 only	97.2 (91.6–100), OU 14 only; 44.8 (38.9–50.7), all OUs	34.4 (17.0–51.8), OU 14 only; 33.5 (27.9–39.1), all OUs
HH with at least one member who participated in MDA and received IRS	–	42.6 (34.2–51.0)	57.3 (49.2–65.3)	44.1 (36.4–51.7)
HH with at least one member who participated in MDA and owns any LLINs (OU 14 only)	–	–	97.2 (91.6–100)	–

CL = confidence limit; HH = household; IRS = indoor residual spraying; LLINs: long-lasting insecticide-treated nets; MDA = mass drug administration; OU = operational unit; T0 = baseline survey; T1 = 10 weeks; T2 = 6 months; T3 = 14 months.

The acceptance of the MDA medications during the campaign was extremely high, at 95.8% of all individuals who agreed to be screened for MDA eligibility (Figure 2). The acceptance of IRS was high as well; 97.2% of the 9,497 households reached and offered IRS accepted the spraying (Abt Associates, IRS campaign monitoring data, not shown). At T1, 99.6% of survey participants who took the MDA medications responded that they would take the medicine again in a future campaign. Among primary respondents who accepted IRS, 85.0% said they would do so again (Table 2).

The coverage indicators were obtained at T1 to minimize the risk of recall bias. The MDA campaign coverage was high at 89.6% among all respondents of the selected households (Table 2). Notably, the coverage estimate by survey confirmed the effective coverage of 90.8% based on the MDA campaign monitoring data (Figure 2).

When asked at T1, 48.9% of primary respondents of households stated their house had received IRS during the campaign. The combined coverage indicator for households with at least one person who took the MDA medication and had their house sprayed was 42.6%. The lower coverage of IRS reflected the percentage of structures constructed of materials suitable for IRS and drove the estimate for the combined indicator. Repeat estimates of the MDA and IRS coverage indicators at T2 and T3 were not statistically different compared with T1 (Table 2).

In the inland OU 14, where LLINs were distributed immediately after T1 instead of IRS, the household ownership of at least one LLIN increased from a baseline (T0) of 3.2% to 97.2% at T2, which was approximately 3 months after LLIN distribution. At T3, the household LLIN coverage fell to 34.4%, which was the same as the coverage across all study areas, highlighting the rapid loss of LLINs by 11 months after distribution (Table 2).

### Feasibility: implementation challenges of MDA/IRS campaign.

Apart from individuals’ acceptance of the campaign interventions, other factors affecting the operational feasibility of reaching high coverage were assessed. The percentage of households reached successfully to receive the interventions was an indicator of feasibility. When asked at T1, 94.8% of primary respondents stated that a team visited their house to offer MDA, whereas the remainder did not receive a visit by the team or did not know. Among 134 individuals (10.4% of all surveyed at T1) who did not take the medications, 61.2% responded they were absent, 17.9% were ineligible/excluded based on the MDA criteria, 14.9% refused the intervention because of personal attitudes related to the intervention, 4.5% refused based on a misunderstanding of the medical exclusions, and 1.5% did not provide a reason (Table 3).

**Table 3:** Indicators of feasibility

Indicator	Survey 1 at 10 weeks, % (95% CL)
Heard about campaign in advance, HH response	96.7 (94.4–99.0)
MDA team reached house to offer meds, HH response	94.8 (91.8–97.8)
Individuals who did not take MDA medications	10.4 (8.0–12.7)
Reasons for not taking medications (*n* = 134; unweighted)	Absent/not living there, 61.2
	Ineligible/excluded, 17.9
	Refused (attitude/perception), 14.9
	Refused (misperception of allowable medical condition), 4.5
	Unspecified, 1.5
IRS team reached house to offer spray (no OU 14), HH response	63.5 (57.4–69.7)
Wall material suitable for IRS (*n* = 257)*	Sprayable, 63.6 (59.4–67.8)
	Could not be sprayed, 36.4 (32.2–40.6)
Eligible houses not sprayed	8.3 (7.7–8.9)
Reasons for not spraying eligible houses (*n* = 788)†	Refused, 33.0%
	Unable to vacate house because of illness, 17.6
	Head of HH unavailable for consent, 16.5
	House closed, no one available, 8.4
	Structure no longer a residence, 3.0
	Miscellaneous or unspecified, 20.8
Both MDA/IRS teams reached house to offer intervention (no OU 14), HH response	59.1 (53.4–64.9)

CL = confidence limit; HH = household; IRS = indoor residual spraying; MDA = mass drug administration; OU = operational unit.

* Estimate collected at 14 months. IRS team reached HH to offer spray: 70.3% (95% CL, 54.2–76.5).

† Data source: Abt Associates, IRS campaign monitoring report, 2018 (unpublished report).

For IRS, 63.5% of primary respondents indicated that a team visited their house and offered IRS. The repeat estimates at T2 and T3 revealed similar results. The suboptimal reach for IRS was a result of the high percentage of houses constructed of materials incompatible with IRS application; 36.4% of houses had walls made of cloth, plastic, or other non-sprayable (unsuitable) material. Of the 9,497 houses that were eligible for IRS, 788 (8.3%) were not sprayed. Of the 788 eligible houses not sprayed, the three main reasons included 33% of households refused IRS, 17.6% could not vacate an ill family member, and 16.5% had a head of household who was not available for consent (Table 3).

### Drug safety: self-reported side effects and PV results associated with MDA medications

Pharmacovigilance conducted between October 9 and December 6, 2018 identified 54 individuals who sought care at health facilities for potential AEs associated with the MDA (Table 4). Of 16 women of childbearing age, one was 7 months pregnant and, appropriately, received only SP during the MDA. She experienced pruritus and asthenia that was assessed as moderate in severity. Others (denominators vary as a result of incomplete data fields) took both SP and PQ. Almost all reported symptoms that were mild and nonspecific. There were three incidents of facial swelling affecting two sisters age 9 and 11 years, and an unrelated 66-year-old woman. The two sisters were treated at the health facility with corticosteroid injections that resolved the symptoms. The third individual’s symptoms resolved after 1 day without treatment. Based on the date of taking the MDA medications, the date of the clinic visit, and specific symptoms, 6% (*n *= 3) were classified as “definite relationship,” whereas most other AE cases were “possible temporal relationship.” Of the 40 reports with completed data fields for symptom severity, 60% were classified as “mild” in severity (does not interfere with daily activities), 37.5% as “moderate” (may interfere with daily activities), and one AE was classified as “severe” (prevents normal daily activities). Upon further investigation, the severe AE was thought to be caused by infectious gastroenteritis, not the MDA medications. There were no reports of hospitalizations, clinical hemolysis (i.e., dark urine), severe rash, or Stevens–Johnson syndrome during the protocol-designated PV period.

**Table 4:** Characteristics of AEs identified by PV or at the T1 survey

Descriptor	PV	Descriptor	T1
No. of individuals experiencing AEs after MDA, detected at health facilities	54	No. of individuals who took MDA medications reporting AEs at survey T1	106
AEs among 36,338 treated by MDA, %	0.15	AEs among 1,183 respondents treated by MDA, % (95% CL)	9.1 (6.95–11.23)
Median age, years (range) (*n* = 53*)	30 (9 months–78 years)	Median age, years (range)	29.5 (1–90)
Female, %	68.5	Female, %	69.8
Took both SP + SLD PQ vs. SP only, % (*n* = 52)	98	NA	NA
Houses that received IRS when AE was reported, *n *(%) (*n* = 42*)	13 (31.0)	NA	NA
Relationship of symptoms to MDA, *n *(%) (*n* = 50*)	Definitely not related, 1 (2)	NA	NA
	Possible temporal relationship, 46 (9%)		
	Definite relationship, 3 (6)		
Severity of symptoms, *n *(%) (*n* = 40*)	Mild, 24 (60)	NA	NA
	Moderate, 15 (37.5)		
	Severe, 1 (2.5)		
Symptoms reported, %	Headache, 64.8	Symptoms reported, %	Dizziness, 62.3
	Weakness/asthenia, 51.9		Fatigue, 19.8
	Gastroesophageal reflux/dyspepsia, 35.2		Nausea/abdominal pain, 11.3
	Subjective fever, 31.5		Subjective fever, 9.4
	Abdominal pain, 31		Headache/body pain, 8.5
	Dizziness, 31.5		Diarrhea, 7.5
	Myalgia, 25.9		Insomnia, 5.7
	Cough, 24.1		Rash, 4.7
	Nausea, 24.1		Throat pain, 1.9
	Pruritus, 22.2		Vomiting, 1.9
	Loss of appetite, 13.0		Facial edema, < 1
	Palpitations, 7.4		Leg weakness, < 1
	Vomiting, 7.4		
	Chills, 5.6		
	Breathing difficulty, 5.6		
	Mouth/facial edema, 5.6		
	Gait disturbance, 3.7		
	Visual changes, 3.7		
	Diarrhea, 1.9		

AEs = adverse events; CL = confidence limit; MDA = mass drug administration; NA = not applicable; PV = pharmacovigilance; SLD PQ = single low-dose primaquine; SP = sulfadoxine–pyrimethamine; T1 = 10 weeks.

* Number of individuals with data field completed if differs from the total of 54 (for PV) or 106 (for T1).

When surveyed at T1 after the campaign, 106 of 1,183 survey respondents (9.1%) who took the MDA medications reported experiencing side effects (Table 4). Of the 106 respondents with side effects, 103 stated they would take the medications again in a future MDA campaign. The three individuals who would not do so again complained of a combination of dizziness, fever, gastrointestinal symptoms, fatigue, insomnia, and headache or body pain.

### Effectiveness: changes in parasite prevalence and antibody response prevalence

Based on the expected prophylactic duration of 30 days for SP in preventing malaria parasitemia, the primary indicator for effectiveness by parasite prevalence was assessed at the earliest follow-up survey time point, T1.^34^ All surveys collected additional biomarkers for serological measures that reflect cumulative exposure to *P. falciparum*. See Table 5 for point estimates and corresponding 95% CLs for results of all survey biomarkers.

**Table 5:** Survey biomarker results

Survey	Sample size, *n*	Seasonality	RDT positive, % (95% CL)	Antigen or antibody positive by multiplex assay	% (95% CL)
Baseline (T0)	1,196	Beginning of high transmission	1.43 (0.71–2.15)	HRP2	1.60 (0.81–2.38)
				Etramp 5 ag 1	10.88 (8.78–12.97)
				AMA1/MSP1	59.53 (56.04–63.01)
Post-campaign 10 weeks (T1)	1,365	End of high transmission	1.31 (0.14–2.48)	HRP2	1.35 (0.17–2.52)
				Etramp 5 ag 1	14.44 (12.06–16.82)
				AMA1/MSP1	64.24 (61.17–67.32)
Post-campaign 6 months (T2)	1,221	Low transmission	0.28* (0–0.61)	HRP2	0.28* (0–0.61)
				Etramp 5 ag 1	18.05* (15.38–20.72)
				AMA1/MSP1	65.88 (62.36–69.39)
Post-campaign 14 months (T3)	1,123	High transmission	2.52 (1.07–3.97)	HRP2	5.65* (3.32–7.99)
				Etramp 5 ag 1	11.84 (9.37–14.30)
				AMA1/MSP1	61.46 (57.60–65.32)

AMA1 = apical membrane antigen 1; CL = confidence limit; Etramp 5 ag 1 = early transcribed membrane protein 5 antigen 1; HRP2 = histidine-rich protein 2; MSP1 = merozoite surface protein 1; RDT = rapid diagnostic test; T0 = baseline survey; T1 = survey at 10 weeks; T2 = survey at 6 months; T3 = survey at 14 months.

* Value is significantly different (*P* < 0.05) compared with baseline.

**Figure 4.**
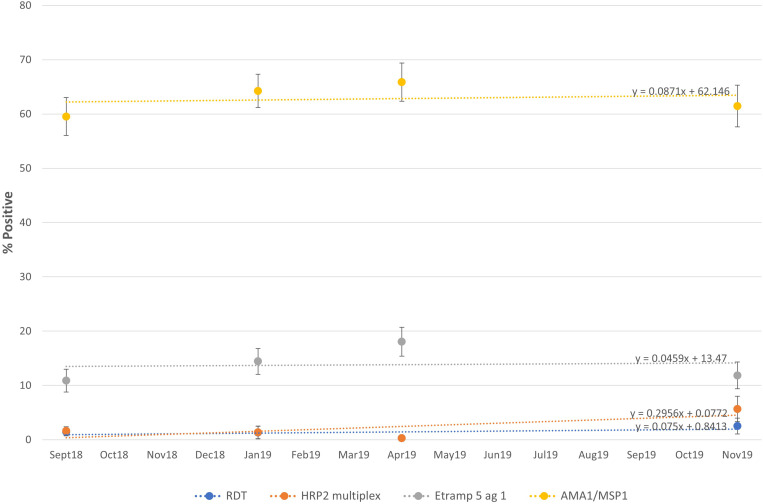
Prevalence with 95% confidence limits and trend lines for the different biomarkers were collected at each survey time point. Above each dotted trend line is the corresponding equation. AMA1 = apical membrane antigen 1; Etramp 5 ag1 = early transcribed membrane protein 5 antigen 1; HRP2 = histidine-rich protein 2; MSP1 = merozoite surface protein 1; RDT = rapid diagnostic test.

At T1, there was no significant difference in parasite prevalence by RDT (1.43% at T0 versus 1.31% at T1) or HRP2 concentration detected by multiplex assay compared with the baseline, T0 (Figure 4). The trend for HRP2 prevalence (by both RDT and multiplex assay) reaches a significantly reduced level of 0.28% at T2 during the dry season, then increased to 2.52% and 5.65% by RDT and multiplex assay, respectively, at T3. Notably, at T3 only, the parasite prevalence detected by multiplex assay is nearly double that detected by RDT.

Seropositivity to *P. falciparum *markers for all survey time points was able to approximate short-term exposure to the parasite in the past 6 months (Etramp 5 ag 1) or long-term exposure to the parasite within the past few years (AMA1/MSP1). The results for Etramp 5 ag 1 at T1 compared with T0 show an increase in seroprevalence from 10.88% to 14.44%, although the difference does not reach statistical significance. The trend for Etramp 5 ag 1 continues to increase to 18.05% at T2, which reflects the cumulative *P. falciparum *exposure, including the recent malaria transmission season. By the next malaria high-transmission season, corresponding to T3, Etramp 5 ag 1 decreased to 11.84%—a level similar to baseline. The longer term antibody responses marked by AMA1/MSP1 show a much higher seroprevalence level (range, 59.53%–65.88%), but a similar trend as seen with the other biomarkers. However, AMA1/MSP1 estimates are within a narrow range and differences between time points are not significant (Figure 4).

## DISCUSSION

In our pilot study, we found a high acceptability of a door-to-door MDA/IRS campaign in Haiti for the goal of malaria elimination. Corroborating the results from a previous formative research study,^35^ community residents were willing to take medication and allow spray teams into their home, despite not feeling ill. Both the expected acceptance of MDA/IRS before the campaign and the actual acceptance of the interventions were high, suggesting that in this context the stated acceptability is a reliable indicator for actual acceptance. Ten weeks after the campaign, the high acceptability persisted, with the same percentage of people expressing their willingness to take medication in a future MDA campaign, but an approximate 15% attrition for IRS acceptance. Because multiple rounds of MDA/IRS would be needed over years to reach elimination, repeated high acceptance of the campaign interventions is a key factor.^36^ This pilot study provides encouragement that, for MDA for malaria elimination, repeated campaigns would be acceptable to the community residents in Haiti, at least during the initial rounds. Recent experience with MDA campaigns for lymphatic filariasis in metropolitan Port-au-Prince has documented declining acceptance and coverage from annual MDA campaigns over a 6-year period.^37^ Anticipating that declining acceptance is likely to occur, developing a framework to both monitor and mitigate this effect would be advantageous to reaching high coverage levels over multiple years. As for IRS, further analysis for the causes of attrition of participants is essential before future spraying.

It was feasible to implement a door-to-door MDA over 4 weeks and IRS over 2.5 weeks to reach an overall population of 40,019 people across small urban and rural settings, and from coastal to mountainous terrain. There were a few operational challenges to achieving maximum coverage. Of the 10.4% who did not take the MDA medications, the majority (61.2%) were absent or did not live there during the campaign. The campaign teams attempted to schedule return visits to provide treatment to anyone traveling and who would return home within 30 days. If capturing those who were not present (either initially or at the time of the return visit) for MDA is needed to reach elimination, a strategy that uses community health workers to provide treatments to returning travelers and new residents could be beneficial. A targeted approach to reach an estimated 2,547 missed individuals (survey data) would likely be more cost-effective than another MDA round and would potentially prevent a community’s fatigue with the program.^37^ Another challenge to deploying a medication-based intervention is that a certain percentage of the population will have a medical contraindication (e.g., allergy, pregnancy, taking another medicine that could interact). In our study, only 1.9% of people surveyed (or 17.9% of those who did not take medications) stated they were willing to take the medication but were ineligible. This percentage could vary depending on population genetics, demographics, and other factors, such as socioeconomic context and access to other medications. Approximately 2.0% of people surveyed (or 19.4% of those who did not take medications) refused the medications because of personal beliefs, attitudes, perceptions, and, some, based on misinformation. Although the refusal rate was low, improvement could be achieved with additional educational messaging, in particular to address misinformation about ineligible health conditions.

As expected, there were more challenges to the IRS intervention related to feasibility. The inland OU 14 was not amenable to IRS because of the rugged terrain and remote location. Instead, LLINs were distributed to each household, which boosted household net ownership dramatically. Our experience demonstrated that LLIN distribution is a feasible option when IRS cannot be implemented. However, the LLIN distribution was delayed by approximately 1 month because of the added complexity of coordinating an additional small campaign and exceeding the absorptive capacity of our teams and partners. Another concern regarding the incorporation of LLINs was the rapid attrition (decrease of 62.8% for households that own any bed net) by 11 months after distribution. The factors contributing to this decrease are unclear; however, it should be noted that the sample size was relatively small, resulting in a wide CI for the estimate of 34.4% household ownership at T3 (95% CI, 17.0–51.8). A study from Ethiopia^38^ found that LLIN attrition was similarly high at 66.3% at 12 months; the main reason for the decrease was that the bed net was thrown away because it was torn or damaged. If LLINs are used as the primary vector control intervention for malaria elimination, future campaigns in Haiti need to assess the factors leading to high attrition.

Our pilot campaign deployed IRS as the primary, targeted vector control intervention that was expected to provide additional protection because most houses received LLINs during a 2017 mass LLIN distribution. In addition, IRS was selected for the intervention because it offered the advantages of less reliance on daily human behavior and the potential benefit of a larger excito-repellency effect against mosquitoes.^39^ Another notable issue limiting IRS coverage was that 36.4% of residential structures were constructed of materials that could not be sprayed. Two years earlier, Hurricane Matthew made landfall in this area of Haiti, causing significant destruction.^40^ It is possible that the high number of structures built of temporary materials is an aftereffect of the natural disaster and not a typical context. Incorporating LLIN delivery to households that cannot be sprayed is an option. The additional operational complexity and anticipation that LLIN coverage will fall within 1 year (as was observed in OU 14) should be considered during strategy planning.

As a standard component of an MDA campaign, our pilot study implemented a passive PV system based at existing health facilities with their available staff. Both SP and PQ have been used in Haiti for many years and are generally considered to be well tolerated, especially at the limited dosing regimen (SP dose: 25/1.25–33/1.7 mg/kg; maximum, 1,500/75 mg; PQ dose: 0.25 mg/kg; maximum, 15 mg) administered during the MDA. The main AEs of concern with SP and SLD PQ are severe cutaneous adverse reactions (SCARs) and acute hemolysis, respectively.^41^^–^^43^ No symptoms consistent with these AEs were reported. Although passive surveillance tends to underreport the true number of events, the symptoms of these severe reactions are so striking that it is unlikely they were missed in our study. In addition, the survey at T1 collected self-reported AEs and similarly did not find symptoms consistent with SCARs or acute hemolysis. However, we include an important addendum here regarding AEs. Based on our 2018 pilot campaign, the Ministry of Health, with Malaria Zero support, implemented an MDA campaign in October 2020 to mitigate the upsurge of malaria cases exacerbated by political unrest and the COVID-19 pandemic that compromised health systems. Four cases of SCARs that were clinically consistent with Stevens–Johnson syndrome, a potentially fatal immune-mediated reaction, were identified among approximately 42,000 people administered SP and SLD PQ over a 5-week period. Three of the affected individuals required hospitalization and all survived. The cause of this startling number of SCARs is not definitive; however, the main differences between our pilot experience in 2018 and the MDA in 2020 were related to the contextual factors of the COVID-19 pandemic.^44^

The effectiveness results are intended to be interpreted in the context of what is known from randomized, controlled trials of MDA—that in areas with low to very low endemicity, a short-term reduction in parasite prevalence has been found within the initial 3 months, but thereafter there was no evidence of a sustained effect.^11^ Our pilot study sought to develop an optimized campaign and then measure the impact in a manner that is typically performed by national programs (i.e., pre- and postintervention comparisons). In addition, the surveys were needed to collect key indicators for feasibility, acceptability, and safety analyses. As an antigen of *P. falciparum *blood-stage infection, HRP2 can persist in the blood typically up to 2 weeks after successful antimalarial treatment.^45^ At T3 only, we found a greater prevalence of HRP2 by multiplex assay compared with detection by RDTs. This finding is a result of the greater sensitivity of the multiplex assay in detecting lower levels of HRP2 that can occur with recently resolved infections or low-parasite density infections (e.g., new, early infections), and is consistent with the timing of the survey in the middle of high transmission.^46^ Alternatively, the absence of this effect, resulting in similar HRP2 levels by multiplex assay and RDTs in the other surveys, may be consistent with lower transmission, but it is difficult to determine because of the low prevalence. The stable parasite prevalence (by HRP2 measured by RDT and multiplex assay) from T0 to T1 may be consistent with a possible, temporary blunting of the peak transmission, as suggested by another study^47^ that showed a reduction in prevalence among easy-access group participants at approximately 4 weeks. However, with seasonal and annual variation of malaria transmission, and in the context of very low transmission, this is difficult to conclude definitively. The 4-week delay in T1 may have placed the data collection closer to the end of the transmission season, when rainfall is less predictable and when fewer infections would naturally be expected. The addition of Etramp 5 ag 1, which measures short-term exposure, was intended to increase the ability to detect a change in transmission by reflecting the cumulative infections over several months and, thereby, enhance statistical power for the comparison. The trend of Etramp 5 ag 1 increased steadily from T0 to T2 without evidence that transmission had decreased. It is likely that antibodies to Etramp 5 ag 1, when used to assess change in malaria transmission at 10 weeks and 6 months after the intervention, have a longer half-life than is suitable. This experience suggests that another monitoring framework or novel tools to assess campaign effectiveness are needed for national programs to measure progress toward elimination. It is also possible that the effectiveness of the combined campaign was diminished by the low coverage of IRS along with low bed net coverage despite a mass LLIN distribution in 2017.

A notable limitation of our study was the lack of a nonintervention, contemporaneous comparison group to assess effectiveness. Our pilot study was not intended to be a controlled trial because numerous randomized, controlled trials have been completed.^11^ In addition, the communities that were selected for the intervention had the highest malaria transmission in the department, which thereby limited the availability of comparison communities (nonintervention communities would have lower malaria transmission). The use of a pre- and post-design was a compromise between “monitoring implementation” and “evaluating effectiveness and its duration” in a manner that many malaria programs are accustomed to deploying.

In summary, our pilot study showed that an MDA/IRS campaign in Haiti is an acceptable and feasible strategy to implement under prevailing conditions. The use of a single-encounter medication regimen (SP and SLD PQ) likely contributed to the high coverage of MDA, whereas IRS coverage was limited by the number of houses that could not be sprayed. The distribution of LLINs instead of IRS offered logistical advantages; however, repeat LLIN distribution is needed with each MDA campaign to mitigate an expected decrease in LLIN coverage between 4 and 11 months. There were no severe AEs identified that were attributable to SP or SLD PQ administered during the 2018 pilot campaign. However, a similar MDA campaign in 2020 identified four cases of SCARs, which raises concerns about using SP for MDA during the current pandemic.^44^ Last, there was no change in parasite prevalence or seroprevalence at 10 weeks after the campaign compared with baseline; however, this finding could be consistent with a temporary blunting of the peak transmission that was not captured between these survey time points. The other possibilities for the lack of a significant difference in parasite prevalence from baseline to 10 weeks could have been the suboptimal IRS and LLIN coverage or the limitations of a pre- and postintervention comparison. For future malaria elimination strategies in Haiti and elsewhere, the experience and results of our pilot study provide a framework for incorporating a targeted MDA/IRS campaign within a package of interventions. The decision to adopt MDA/IRS is not an easy one because of the limited duration of an effect, and reaching malaria elimination requires a commitment to multiple rounds. If national malaria programs decide to incorporate these interventions, either for elimination or as a response to an outbreak, the considerations and lessons learned in our pilot study may aid in optimizing their strategy.

## Financial Disclosure

Funding was provided by the U.S. Centers for Disease Control and Prevention and by a grant from the Bill & Melinda Gates Foundation (OPP1114297) to the CDC Foundation as part of the Malaria Zero Consortium.
